# Neuron-Specific Enolase, S100 Calcium-Binding Protein B, and Heat Shock Protein 70 Levels in Patients With Intracranial Hemorrhage: Erratum

**DOI:** 10.1097/01.md.0000476046.44577.d5

**Published:** 2015-12-18

In the article “Neuron-Specific Enolase, S100 Calcium-Binding Protein B, and Heat Shock Protein 70 Levels in Patients With Intracranial Hemorrhage”,^1^ which appeared in Volume 94, Issue 45 of *Medicine*, Dr. Evren Ekingen's name was omitted from the byline. The article has since been corrected online.
